# The usefulness of small-area-based socioeconomic characteristics in assessing the treatment outcomes of type 2 diabetes patients: a register-based mixed-effect study

**DOI:** 10.1186/s12889-018-6165-3

**Published:** 2018-11-14

**Authors:** Maija Toivakka, Aki Pihlapuro, Markku Tykkyläinen, Lauri Mehtätalo, Tiina Laatikainen

**Affiliations:** 1 Department of Geographical and Historical Studies, University of Eastern Finland, P.O. Box 111, FI-80101 Joensuu, Finland; 2 Joint municipal authority for North Karelia social and health services (Siun sote), Tikkamäentie 16, FI-80210 Joensuu, Finland; 3 School of Computing, University of Eastern Finland, P.O. Box 111, FI-80101 Joensuu, Finland; 4 Institute of Public Health and Clinical Nutrition, University of Eastern Finland, P.O. Box 1627, FI-70211 Kuopio, Finland; 5 Department of Public Health Solutions, National Institute for Health and Welfare (THL), P.O. Box 30, FI-00271 Helsinki, Finland

**Keywords:** Individual-level socioeconomic status, Small-area-based socioeconomic status, Care outcomes, Type 2 diabetes mellitus, Electronic health records

## Abstract

**Background:**

Assessment of the differences in the outcomes of care by socioeconomic status (SES) is beneficial for both the efficient targeting of health care services and to decrease health inequalities. This study compares the effects of three patient-based SES predictors (earned income, educational attainment, employment status) with three small-area-based SES predictors (median income, educational attainment, proportion of the unemployed) on the treatment outcomes of type 2 diabetes patients.

**Methods:**

Mixed-effect modeling was applied to analyse how SES factors affect the treatment outcomes of type 2 diabetes patients. The treatment outcomes were assessed by the patients’ latest available glycated hemoglobin A_1C_ (HbA1c) value. We used electronic health records of type 2 diabetes patients from the regional electronic patient database, the patients’ individual register-based SES information from Statistics Finland, and the SES information about the population of the postal code area of the patients from Statistics Finland.

**Results:**

The effects of attained education on the treatment outcomes, both at the patient-level and the small-area-level are quite similar. Age and male gender were associated with higher HbA1c values and lower education indicated higher HbA1c values. Unemployment was not associated with HbA1c values at either the patient-level or the area-level. Income gave divergent results: high values of HbA1c were associated with low patient incomes but the median income of the postal code area did not predict the treatment outcomes of patients.

**Conclusions:**

Our comparative study of three SES factors shows that the effects of attained education on the treatment outcomes are rather similar, regardless of whether patient-based or small-area-based predictors are used. Small-area-based SES variables can be a good way to overcome the absence of individual SES information, but further research is needed to find the valid small-area factors by disease. This possibility of using more small-area-based data would be valuable in health service research and first-hand planning of health care services.

## Background

Individual-level and area-based socioeconomic status (SES), such as income, education and occupation, have been used to examine the associations between SES and health risks in chronic disease patients. For example, previous research has shown that low individual or neighbourhood SES is associated with the risk of getting diabetes [1–3], the increased prevalence of chronic obstructive airway diseases [4], all-cause mortality in adults with atrial fibrillation [5] and increased risk of coronary heart disease [6–8]. In addition, the care of diabetes can be influenced by individual and neighbourhood SES [9, 10].

The patient’s SES information is rarely linked to public health databases or patient medical records. Thus, if the impacts of individual SES factors on care outcomes are to be assessed, then it is necessary to conduct surveys or combine information from other databases (e.g., census, educational, occupational, housing and tax records), which may not be easily accessed. Access to individual SES information often requires a cumbersome permission processes due to the need to ensure information security, which consumes time and money. Socioeconomic variables by area are widely used in health research [2, 3, 5, 6, 8] and this has been suggested as a sufficiently valid and easy approach to overcome the absence of individual SES information [11, 12].

The aim of this study is to compare the predictive values of patients’ individual SES variables with the respective SES variables of postal code areas on the treatment outcomes of type 2 diabetes patients. The treatment outcomes were assessed by the patients’ latest available glycated hemoglobin A_1C_ (HbA1c) value, which was used as an indicator of good glycemic control. We investigated whether the socioeconomic characteristics of patients are overwhelmingly more meaningful than respective SES variables of postal code areas or if they both provide similar predictive results about the influence of SES on the treatment outcomes. If the small-area-based average of SES has a predictive value, then it could be used in first-hand planning and targeting of health care services.

## Methods

### Patient group and glycemic control

In this study, the data consists of all diagnosed type 2 diabetes (ICD10 code E11) patients (10,204) at the end of 2012 in the region of North Karelia (13 municipalities, 165,800 inhabitants), Finland. The prevalence of type 2 diabetes in the population was 6.2% in 2012. The patient data is retrieved from the regional electronic patient database and the use of the data was approved by the ethics committee of the North Savo Hospital District. The data have a nested grouping structure with 13 municipalities, 131 postal code areas (4–33 postal code areas per municipality) and 10,204 patients, out of which 10,067 patients were able to have their postal code of residence identified (5–623 patients per postal code area).

The treatment outcomes were assessed by the patients’ latest available glycated hemoglobin A_1C_ (HbA1c) value in the time period from 3.1.2011–16.1.2013. HbA1c provides a long-term blood sugar value and it was used as an indicator of good glycemic control. The recommended HbA1c level for good treatment balance is < 7% (53 mmol/mol) based on Finnish guidelines but also according to the American Diabetes Association (ADA) standards of medical care HbA1c < 7% is a reasonable goal for many adults [13]. Altogether, HbA1c measurement was found for 89.9% (*n* = 9172) of the patients. Out of these patients, 72.5% (*n* = 6652) reached the recommended HbA1c level. The average HbA1c value was 6.6 (Table 1).

**Table 1:** Statistical characteristics for HbA1c value, patient-based and small-area-based data

Variable	N	Mean/Proportion	SD	Min.	Max.
HbA1c	9172	6.6	1.2	4.1	15.3
Gender	10,204				
Male	5402	52.9			
Female	4802	47.1			
Age (patient)	10,204	67.9	12.1	13	99
Income (patient)	10,173	18,409.5	12,180.9	N/A	N/A
Education (patient)	10,204				
No degree	5190	50.9			
Upper secondary level education	3609	35.4			
Lowest level tertiary education	781	7.7			
Lower-degree level tertiary education	374	3.7			
Higher-degree level tertiary education	216	2.1			
Doctorate level tertiary education	34	0.3			
Unemployed (patient)	432	4.2			
Median income (small-area)	131	16,083.0	3146.7	10,251	24,417
Educated, % (small-area)	131	62.3	9.9	38.4	84.5
Unemployed, % (small-area)	131	7.2	2.3	3.0	14.4

### Patient-based predictors

Each patient’s age, gender, earned income (€), educational attainment and employment status were used in the analysis (Table 1). The patient’s age and gender were obtained from the electronic patient database and the socioeconomic characteristics of each patient were provided by Statistics Finland via its protected remote access service, confidentially according to the Personal Data Act. Individual socioeconomic characteristics from Statistics Finland are from the end of the year 2012. Education was based on the patient’s latest highest degree and it was classified into six classes: no degree, upper secondary level education, lowest level tertiary education, lower-degree level tertiary education, higher-degree level tertiary education, and doctorate or equivalent level tertiary education. The information on whether the patient is unemployed was retrieved from Statistics Finland’s main type of activity variable. ‘Main type of activity’ describes the nature of a person’s economic activity during a year.

### Small-area predictors

To measure the role of neighbourhood in the treatment outcomes, small-area-based socioeconomic variables were gathered from the 2011 Statistics Finland postal code area database. Three variables were used to describe the socioeconomic characteristics of the postal code areas: median income, the proportion of people with at least a high school diploma or vocational training, and the proportion of people unemployed (Table 1). These three variables were selected to test the predictive value of small-area-based variables for the treatment outcomes because we had patient-based corresponding variables for comparison.

### Analyses

To analyse how the SES variables at the level of single patient, postal code area and municipality affect the treatment outcome of the type 2 diabetes patients, we used the following mixed-effect model with a random intercept:

$$ {y}_{ij k}=\boldsymbol{\beta} {\prime}_P{\boldsymbol{x}}_{ij}^{(P)}+\boldsymbol{\beta} {\prime}_I{\boldsymbol{x}}_{ij k}^{(I)}+{b}_i^{(M)}+{b}_{ij}^{(P)}+{e}_{ij k} $$

where *y*_*ijk*_ is the HbA1c value of the patient *k* of postal code area *j* within municipality *i,* ${\boldsymbol{x}}_{ij}^{(P)}$ includes the postal code area predictors and ***β***′_*P*_ the corresponding regression coefficients, ${\boldsymbol{x}}_{ijk}^{(I)}$ includes the patient-based predictors and ***β***′_*I*_ the patient-based regression coefficients, ${b}_i^{(M)}$ is the random effect for municipality, *i,*${b}_{ij}^{(P)}$ is the random effect for postal code area *j* within municipality *i*, and *e*_*ijk*_ is the residual error of patient *k* in postal code area *j* of municipality *i*. The random effects and residuals are assumed to be independent and normally distributed with zero means and variances ${\sigma}_M^2$, ${\sigma}_P^2$, and *σ*^2^. The random effect is used to take into account the grouped, nested structure of the data [14]. More specifically, parameter ${\sigma}_M^2$ describes the unexplained variability in the municipality-level means of HbA1c, ${\sigma}_P^2$ correspondingly describes the unexplained variability of postal code area-based means around the municipality-level mean, and residual variance *σ*^2^ describes the unexplained variability of individual observations around the postal code area-based mean. At the same time, they model the dependence of observations that belong to the same postal code area or municipality, thus allowing hypothesis testing on the fixed effects that takes into account the lack of independence among the observations from the same groups. Because the variance components are independent, the variances can be directly summed to obtain *unexplained area-based variance* as ${\sigma}_M^2+{\sigma}_P^2$ and *total unexplained variance* as ${\sigma}_M^2+{\sigma}_P^2+{\sigma}^2$, and the corresponding standard errors as a square root of the variance. We also considered more advanced mixed-effect models with random intercept and slope, but the model with random intercept was deemed sufficient.

Several models were fitted to the dataset. The first model, the *simple model* (SM) included only a fixed intercept, age, gender and the random effects and residuals, providing estimates of the total variability among municipalities, postal code areas, and patients within postal code areas. The other models included additional patient-based fixed predictors (*patient-based model*, PBM), small-area-based predictors (*area-based model*, ABM) and both (*combined model*, CM). By comparing the estimated variances of random effects among these models, we analysed the potential of the small-area-based and patient-based predictors in explaining the variability in HbA1c. We were especially interested in whether the patient-based models or combined models had much lower total unexplained variance (i.e., the sum of the unexplained variability between municipalities, postal code areas, and patients) than the area-based model.

## Results

Adding the small-area-based or patient-based socioeconomic variables to the simple model reduces the total unexplained variability (Table 2, Random part column), which confirms that there is such a component in the unexplained variability of the simple model that can be explained by the socioeconomic variables. However, the component is small, only 1.2% [(1.2325^2–1.2252^2)/1.2325^2*100% = 1.2%] compared with the total unexplained variability in the simple model but 47% [(0.1473^2–0.1076^2)/0.1473^2*100% = 47%] compared with the total unexplained variability at the area-level. The small-area predictors in the area-based model reduce the area-based unexplained variability compared with the simple model, whereas the patient-based predictors in the patient-based model explain both patient-based variability and area-based variability. Interestingly, adding the patient-based predictors to the area-based model (combined model) provides only very slight (0.3% [(1.2252^2–1.2232^2)/1.2252^2*100% = 0.3%]) reduction to the total unexplained variability compared with the area-based model. This confirms that the small-area predictors alone can explain a major part of such variability in the HbA1c that is associated with the socioeconomic factors, while in comparison, patient-based information provides only a slight improvement.

**Table 2:** Parameter estimates for simple model (SM), patient-based model (PBM), area-based model (ABM), and combined model (CM)

	SM		PBM		ABM		CM	
			Fixed part^a^					
Variable	Estimate (95% CI)	*p*-value	Estimate (95% CI)	*p*-value	Estimate (95% CI)	*p*-value	Estimate (95% CI)	*p*-value
Age	4.86 (2.68–7.04)	0.00	2.98 (0.29–5.67)	0.03	4.97 (2.78–7.15)	0.00	3.06 (0.37–5.74)	0.03
Gender (2 = male)	83.00 (31.95–134.06)	0.00	101.24 (49.16–153.32)	0.00	83.18 (31.95–134.40)	0.00	100.84 (48.80–152.88)	0.00
Income (patient)			−5.24 (−8.01–(−2.46))	0.00			−5.05 (−7.82–(−2.28))	0.00
Education (patient)			−40.18 (−71.80–(− 8.56))	0.00			−35.24 (−66.93–(−3.56))	0.03
Unemployed (patient)			77.54 (−71.57–226.65)	0.08			74.54 (−74.53–223.62)	0.33
Income (small-area)					0.02 (0.00–0.04)	0.08	0.02 (0.00–0.04)	0.10
Education (small-area)					−14.33 (−20.27–(−8.39))	0.00	−12.75 (−18.74–(−6.77))	0.00
Unemployed (small-area)					7.25 (−12.39–26.89)	0.47	4.40 (−15.31–24.12)	0.66
			Random part^b^					
Municipality (M)	0.0859^2^		0.0774^2^		0.0961^2^		0.0944^2^	
Postal code area (Pa)	0.1196^2^		0.1115^2^		0.0483^2^		0.0496^2^	
Patient (P)	1.2237^2^		1.2177^2^		1.2205^2^		1.2185^2^	
Areas (M + Pa)	0.1473^2^		0.1357^2^		0.1076^2^		0.1066^2^	
Total (M + Pa + P)	1.2325^2^		1.2252^2^		1.2252^2^		1.2232^2^	

^a^The fixed part presents estimated regression coefficients of the linear mixed-effect models and their 95% Confidence Intervals (95% CI) and p-values. The estimates and 95% Confidence Intervals have been multiplied by 1000 to help the interpretation process

^b^The random part presents unexplained variance at different levels (in the form *se*^2^)

The Table 2 fixed-part column describes the estimated regression coefficients of a simple mixed-effect model (SM) on age and gender, patient-based model (PBM) for patient-based predictors, area-based model (ABM) for postal code area predictors, and a combined model (CM) for both. In addition to the patient’s age and male gender, which both increase the HbA1c level, less educated people have a higher HbA1c value. This effect can also be rather well explained by the proportion of people with at least a high school diploma or vocational training by area. When patient-based information on education is not used (ABM), the coefficient of the education at the level of the postal code area increases and models at least part of the variation, which is modeled through patient-based education in PBM and CM. A comparison of the coefficient of small-area-based education 14.33*10^− 3^ to the minimum and maximum education proportions in the data (0.384–0.845 Table 1) shows that it can at most explain about 0.007 unit differences in the mean HbA1c value between postal code areas, which is about 8% (0.007/0.08318*100% = 8%) of the difference between the genders. The conclusion on the effects of educational factors is that either patient-based or small-area-based factors have quite similar impacts. The patient’s income is also a significant predictor in PBM and CM, showing that high values of HbA1c are associated with low incomes, but this association is not present at the ABM. Unemployment does not have an effect on the HbA1c value of either the patient-level or area-level.

## Discussion

In this study, we used electronic health records about type 2 diabetes patients from the regional electronic patient database, the patient’s individual register-based SES information and register-based SES information by postal code area to compare the effect of patient-based and small-area-based factors of SES on the treatment outcomes. Patients’ glycemic control was used as an example of treatment outcome. We tested how the patient’s HbA1c value is associated with different patient-based and postal code area SES factors.

In these analyses, age and male gender were associated with higher HbA1c values and less educated patients had a higher HbA1c value, as did those living in low-educated areas. Unemployment did not have an effect on the HbA1c value of either the patient-level or small-area-level. Income was the only predictor that gave divergent results: high values of HbA1c were associated with patients’ low incomes, but these associations were not present at the small-area-level.

Multilevel analysis revealed that the educational attainment of a neighbourhood amidst the area-based socioeconomic variables can explain a major part of such variability in the HbA1c that is associated with socioeconomic characteristics of a neighbourhood, while in comparison patient-based information on SES provides only a slight improvement. This means that the small-area-based information on educational attainment can be almost as useful as patient-based information when assessing the socioeconomic differences in the treatment outcomes.

There has been previous research with similar and conflicting results on the agreement between individual-level and area-based SES factors [11, 12, 15, 16]. However, this previous research has focused on health outcomes, health inequalities, or health risk factors but not on the treatment outcomes. For example, Krieger [11] compared the association of individual-level and census-based socioeconomic variables with hypertension, height, smoking, and number of full-term pregnancies. He concludes that the methodology provides a valid and useful approach to overcoming the absence of individual socioeconomic data. Domínguez-Berjón et al. [12] investigated the association between health outcomes (perceived health status, the presence of at least one chronic condition, smoking) and small-area-based socioeconomic measures, and also the association with individual socioeconomic measures. Both yielded similar results and they conclude that area-based measures can be applied to monitor health inequalities when individual information is not available. Marra et al. [15] determined the agreement between aggregate-level and individual SES factors among asthma, diabetes, and rheumatoid patients. They found that agreement between individual-level and aggregate-level SES variables may depend on patient group and in their study, individual-level variables were assumed to be better than aggregate-level variables. Pardo-Crespo et al. [16] studied the agreement between individual and area-level SES measures and compared the association of individual- and area-level SES measures with health outcomes (low birth weight, childhood obesity, and smoking household members) among children. They found that there was a significant disagreement between individual-level and area-level SES measures. However, these previous studies have been mainly correlative and they have not used mixed-effect models to test the explanatory power of SES variables.

In our study, we used mixed-effects models to take into account the nested grouped structure of the data into municipalities and postal code areas within municipalities. This allowed us to analyse which components of the total variability were explained by the small-area-based and patient-based predictors. It also took the dependence of the data into account in the tests of the fixed predictors. Ignoring the dependence by treating each patient as an independent observation would have led to an anti-conservative test (too small *p*-values) in this situation.

A strength of this study was that it included all diagnosed cases of type 2 diabetes in the region, eliminating selection bias. In addition, we used objective register-based socioeconomic information both at the patient-level and area-level gathered from Statistics Finland. One limitation of the data is that the regional patient database does not include patient data from private occupational health care. This can actually mitigate the SES differences, as employed patients, most likely, would have even better treatment outcomes. The study did not analyse lifestyles (e.g., nutrition, physical activity) or health care processes. However, these factors are not available in electronic health registers and this can be seen as one serious limitation of register-based studies.

Based on our results, when assessing the treatment outcomes of type 2 diabetes patients, small-area-based SES variables (such as education) can provide a useful way to predict the treatment outcomes by area. We could assume that this assessment method also applies to the care of other chronic conditions, but this would need more research with different patient groups and with different outcome measures. Small-area-based variables can be a good way to overcome the absence of individual SES information, as suggested previously [11, 12], but further research is needed to find more valid area-based factors. Given that individual-level data on socioeconomic characteristics are not easily available and require lengthy and expensive permission processes due to the need to ensure information security, small-area-based SES variables could be more widely used at a low cost.

## Conclusions

In summary, our comparative study of three SES factors shows that the effects of attained education on the treatment outcomes are rather similar, regardless of whether individual or area predictors are used. If it is possible to target health care services on demand by area, then the use of internally valid small-area-based SES factors provides cost-efficient first-hand information for improving quality and equity in health care. This possibility of using more small-area-based data would be valuable in health service research and in planning where large diagnostic-focused patient materials are used, and access to individual-level information on socioeconomic characteristics is complicated and expensive.

## Abbreviations

ABM: Area-based model
CM: Combined model
HbA1c: Glycated hemoglobin A_1C_
PBM: Patient-based model
SES: Socioeconomic status
SM: Simple model
